# The cervicovertebral angle as an associated factor of neck pain in female manufacturing workers: a dual-model cutoff analysis using logistic regression and decision trees

**DOI:** 10.1186/s12891-026-10115-5

**Published:** 2026-06-23

**Authors:** Seung-yoon Han, Ui-jae Hwang, Yoon-ji Lee, Il-kyu Ahn, Yul-ji Won, Ji-hoo Moon, Oh-yun Kwon

**Affiliations:** 1 Department of Physical Therapy, The Graduate School, Yonsei University, Wonju, Gangwon-do South Korea; 2 Department of Physical Therapy, College of Health Sciences, Laboratory of KEMA AI Research (KAIR), Yonsei University, Wonju, South Korea; 3 Department of Physical Therapy, College of Health Sciences, Laboratory of Kinetic Ergocise Based on Movement Analysis, Yonsei University, Wonju, Gangwon-do South Korea; 4 Department of Physical Therapy, College of Health Sciences, Yonsei University, 1, Yeonsedae-gil, Maeji-ri, Heungeop-myeon, Wonju-si, Gangwon-do 26493 South Korea

**Keywords:** Cervicovertebral angle, Forward head posture, Nonspecific neck pain, Logistic regression, Decision tree, Cutoff value, Female workers

## Abstract

**Background:**

Forward head posture is commonly quantified using the cervicovertebral angle (CVA), a simple static postural measure used in clinical and occupational settings. Although reduced CVA has been associated with nonspecific neck pain (NSNP), its discriminative utility as a single postural metric remains uncertain because NSNP is multifactorial. This study aimed to examine the association between CVA and NSNP in female manufacturing workers and to identify exploratory CVA cutoff values using logistic regression (LR) and decision tree (DT) models.

**Methods:**

This cross-sectional study included 135 female manufacturing workers, classified into an NSNP group (*n* = 64) and a non-pain group (*n* = 71). CVA was measured using standardized sagittal-view digital photography and ImageJ software. Unadjusted and adjusted binary LR analyses were performed to examine the association between CVA and NSNP. Receiver operating characteristic curve analysis was used to determine the optimal LR-based cutoff value using the Youden index. A DT algorithm was applied to identify hierarchical cutoff values. Model performance was evaluated using accuracy, sensitivity, specificity, and area under the curve (AUC).

**Results:**

The NSNP group showed a significantly smaller CVA than the non-pain group (51.16° ± 5.47° vs. 53.21° ± 4.11°, *p* = 0.015). In the unadjusted LR model, CVA was significantly associated with NSNP (odds ratio [OR] = 0.914, 95% confidence interval [CI]: 0.849–0.984, *p* = 0.017). This association remained significant after adjustment for age, height, weight, and body mass index (OR = 0.911, 95% CI: 0.830–0.999, *p* = 0.047). The LR model identified an optimal cutoff value of 51.40°, with an AUC of 0.623. The DT model identified a primary cutoff of 51.54° and a secondary threshold of 42.27°, with an AUC of 0.645.

**Conclusions:**

CVA was significantly associated with NSNP in female manufacturing workers, and both models identified a consistent primary cutoff of approximately 51.5°. However, CVA alone showed limited classification ability. Therefore, the identified cutoff values should be interpreted as supplementary screening references.

**Trial registration:**

This study was registered by the Korea Clinical Research Information Service (KCT0011501).

## Background

Forward head posture (FHP) is characterized by an anterior displacement of the head relative to the cervical spine [1]. This postural alteration increases the biomechanical load on cervical structures, which has been associated with muscle tension, ligament stress, and ultimately, the development of chronic neck pain [2, 3]. FHP is characterized by shortened upper cervical extensors and lower cervical flexors [4, 5], a muscular imbalance that has been reported to be related with neck pain. The cervicovertebral angle (CVA) is widely used in clinical practice as a quantitative measure of FHP [6, 7]. Females exhibit a two-times higher prevalence of cervical musculoskeletal disorders than males [8–10]. This difference should be interpreted as multifactorial, reflecting the combined influence of biomechanical factors, psychosocial factors, hormonal influences, and ergonomic and occupational conditions [10–12]. Nonetheless, previous studies have suggested that the factors associated with neck pain may differ by sex. In males, neck pain has been reported to be more associated with factors such as lower trapezius muscle weakness, whereas in females, it may be more related to cervical posture-related factors, including cervical rotation [13]. Therefore, mechanisms responsible for neck pain may differ according to sex, with FHP having more associated with women.

Although CVA is widely used as a simple postural indicator of FHP, it is a static two-dimensional measure and cannot fully capture the three-dimensional biomechanics of the cervical spine [14]. Previous studies have suggested that a CVA of approximately 53° may indicate normal cervical alignment, while a CVA of < 50° has been proposed as indicative of FHP, and values < 44° have been associated with severe FHP [15–17]. Generally, decreases in CVA are related to greater neck pain intensity and higher disability scores, supporting a possible association between neck pain and FHP [18, 19]. However, these CVA thresholds have not been consistently validated, and considerable overlap exists between symptomatic and asymptomatic populations. For instance, one study reported that asymptomatic individuals exhibited a mean CVA of 48.63°, which is within the generally defined FHP range, whereas individuals with neck pain demonstrated a mean CVA of 44.44° [20]. Furthermore, studies in younger individuals or in those not experiencing neck pain have also reported only weak relationships or have failed to identify any significant associations between CVA and neck pain severity [21, 22]. Similarly, previous cluster analysis studies on individuals with nonspecific neck pain (NSNP) found no significant association between static CVA and pain severity [7]. Notably, one subgroup demonstrated the most severe pain despite exhibiting CVA values comparable to those of pain-free individuals [7]. These inconsistencies suggest that the relationship between CVA and NSNP is complex and potentially nonlinear, and that CVA alone cannot be expected to serve as a high-accuracy diagnostic tool for NSNP. Nevertheless, CVA is a simple, low-cost measure that is widely used in clinical and occupational settings for evaluating cervical posture [6, 7]. Although previous studies have reported associations between CVA and neck pain, it remains unclear how CVA values should be interpreted in relation to NSNP. The absence of a statistically derived reference value may limit the consistent interpretation of CVA measurements across research and clinical environments. Therefore, examination of CVA cutoff as an exploratory reference value rather than as a diagnostic criterion for NSNP.

Logistic regression (LR) analysis has traditionally been used as a standard method for determining cutoff values [23]. LR models the log odds of the outcome as a function of predictor variables, allowing the effects of continuous variables to be expressed as odds ratios. Although conventional LR assumes a linear relationship between continuous predictors and the log odds of the outcome, nonlinear relationships can also be accommodated by applying flexible modeling approaches, such as transformed variables, interaction terms, polynomial terms, or spline functions [24, 25]. The decision tree (DT) models are nonparametric models, meaning that they do not assume a linear relationship between variables [26]. Therefore, DT models are useful for determining cutoff values by repeatedly splitting the data, which allows them to capture complex relationships and nonlinear patterns. Accordingly, applying both LR and DT models may provide complementary information regarding the association between CVA and NSNP and the exploratory identification of CVA cutoff values.

However, because NSNP is a multifactorial condition, the predictive performance of CVA as a single postural metric may have some limitation. Therefore, the purpose of this study was to determine whether a statistically meaningful cutoff value exists using both LR and DT models. We hypothesized that CVA would be statistically associated with NSNP. In addition, both models were expected to identify potential threshold values that may provide exploratory clinical interpretation.

## Methods

### Participants

This cross-sectional study recruited 135 female workers from the manufacturing industry from 01/2026 to 02/2026. The sample size was determined using G*Power v. 3.1.9 (Franz Faul, University of Kiel, Kiel, Germany), based on a pilot study (NSNP group = 20, non-pain group = 25) that reported a standardized odds ratio (OR) of 1.70 for LR. Using the baseline prevalence of neck pain in this pilot population (0.44), a power of 0.80, and a significance level of 0.05, the power analysis indicated that a minimum of 129 participants were required for this study.

**Fig. 1:**
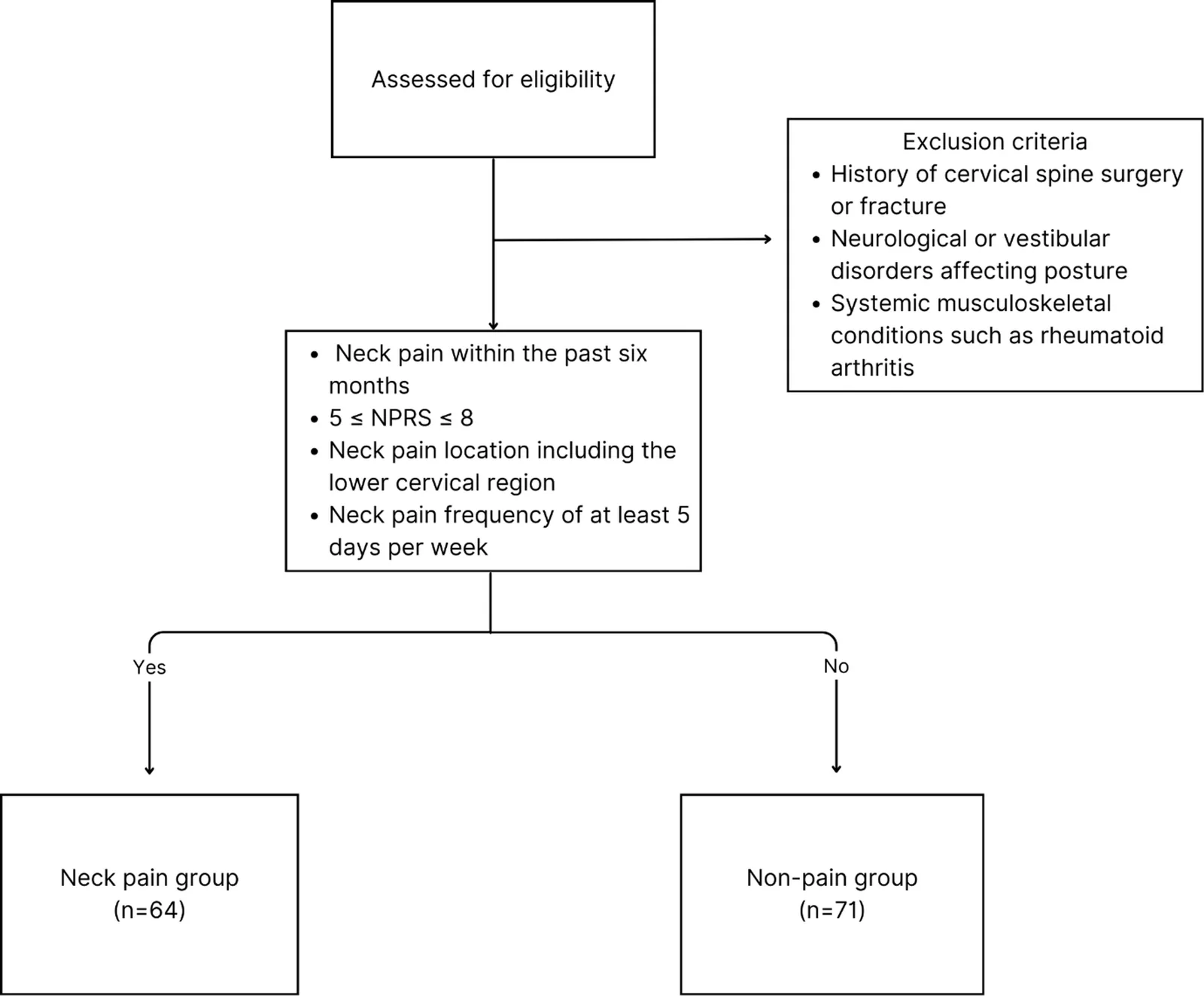
Flow diagram of study participant selection

Figure 1 presents a flowchart outlining the recruitment process used in this study. The inclusion criteria were as follows: (1) age between 18 and 65 years; (2) ability to maintain an upright standing posture; and (3) willingness to provide informed consent. In this study, the NSNP group was defined as individuals with moderate or greater neck pain: (1) individuals who reported neck pain within the past six months, (2) individuals with Numeric Pain Rating Scale (NPRS) scores ranging between ≥5 and ≤ 8 [27], (3) pain location including the lower cervical region, and (4) pain frequency of at least 5 days per week. The non-pain group was defined as individuals who reported no neck pain in the preceding six months and had an NPRS score of 0 at the time of assessment. Based on these criteria, the participants were divided into an NSNP group (*n* = 64) and a non-pain group (*n* = 71). The exclusion criteria were as follows: (1) history of cervical spine surgery or fracture, (2) neurological or vestibular disorders affecting posture, and (3) systemic musculoskeletal conditions such as rheumatoid arthritis.

All participants signed an informed consent form, and the study was approved by the Yonsei University Mirae Campus Institutional Review Board (approval number: 1041849-202512-BM-269-01).

### Instruments

The CVA was measured using a standardized digital photography method. A smartphone (Galaxy S22 Ultra, Samsung Inc., Seoul, Korea) was used to capture sagittal-view photographs [28]. All photographs were analyzed using ImageJ software (v.1.53, National Institutes of Health). The reliability and validity of ImageJ software have been reported previously (intraclass correlation coefficient: 0.92 to 0.99) [29].

### Measurement of CVA

The participants stood in a relaxed, upright position with their feet shoulder-width apart, arms resting naturally at their sides, and their gaze directed forward. Markers with diameters of 20 mm were attached to the spinous process of C7 and the tragus of the ear. Photographs were taken in the sagittal plane at a distance of 1.5 m aligned with the shoulder height of the participant [1]. The CVA was defined as the angle between the horizontal line through C7 and the line connecting C7 to the tragus [1]. The mean values of the two CVA measurements were used for the statistical analysis.

### Statistical analysis

All statistical analyses were performed using SPSS v.28 (IBM Corp., Armonk, NY, USA). The Kolmogorov-Smirnov Z-test was used to assess the assumption of normal distribution. Demographic differences between the groups were analyzed using independent t-tests. For each between group comparison, the t-value, p-value, and effect sizes were calculated using Cohen’s d. Cohen’s d values of 0.10, 0.30, and 0.70 were interpreted as small, moderate, and large effects, respectively [30].

Measurement reliability and measurement error for CVA were assessed using 2-times repeated CVA measurements. Intraclass correlation coefficients (ICCs) with 95% confidence intervals (CIs) were calculated to evaluate measurement reliability. The standard error of measurement (SEM) and the minimal detectable change at the 95% confidence level (MDC95) were calculated.

Binary LR analysis was used to examine whether CVA significantly predicted the presence of neck pain. The OR and 95% confidence interval (CI) were calculated for CVA to estimate the change in the odds of NSNP associated with each one-degree change in CVA. The overall fit of the LR model was assessed using the Hosmer-Lemeshow fit test and the Nagelkerke R² statistic. Receiver operating characteristic (ROC) curve analysis was performed to evaluate the discriminative ability of CVA in classifying individuals with and without neck pain. The area under the curve (AUC) was calculated, with values of 0.5–0.7 considered poor, 0.7–0.8 acceptable, 0.8–0.9 good, and > 0.9 excellent discrimination [31]. Sensitivity, specificity, and optimal cutoff values were determined using the Youden index.

DT analysis was also used to determine the optimal cutoff value of CVA for predicting the presence of neck pain. The classification and regression tree (CRT) algorithm was used, with group (neck pain = 1, no pain = 0) as the dependent variable and CVA as an independent variable. The splitting criterion was based on the Gini impurity index. The maximum tree depth was set to two levels, with a minimum parent node size of 10 cases and a minimum child node size of 5 cases. No pruning was applied, and no internal validation or cross-validation was performed. The resulting classification model was evaluated for predictive performance by calculating classification accuracy, sensitivity, and specificity and by further evaluating the discriminative ability of the model using ROC curve analysis. A significance level was set at *p* < 0.05 for all statistical tests.

## Results

This study included 135 female participants. Participants with NSNP demonstrated significantly higher NPRS scores and NPQ total scores, lower CVA and greater body weight, BMI, and height compared with those without NSNP (Table 1). The CVA measurement demonstrated excellent reliability, with an ICC of 0.983 (95% CI, 0.977–0.988). The SEM and MDC95 were 0.635° and 1.76°, respectively. The approximately 2° between-group difference in CVA slightly exceeded the MDC95, suggesting that this difference was unlikely to be explained solely by measurement error.

**Table 1:** Demographic and general characteristics of the subjects (*N* = 135)

Variable	NSNP group (*n* = 64)	Non-pain group (*n* = 71)	t	*p*-value	Cohen’s d	Interpretation
	Mean	Mean				
CVA (°)	51.16 ± 5.47	53.21 ± 4.11	-0.475	0.015*	-0.427	Moderate
Age (years)	36.20 ± 4.57	36.82 ± 5.35	-0.712	0.478	-0.123	Small
Height (cm)	161.83 ± 4.74	159.69 ± 5.34	2.450	0.016*	0.422	Moderate
Weight (kg)	66.02 ± 16.50	59.41 ± 10.84	2.775	0.008*	0.478	Moderate
BMI (kg/m²)	25.15 ± 5.99	23.27 ± 3.95	2.172	0.036*	0.374	Moderate
Work duration (month)	15.44 ± 4.24	16.04 ± 5.40	-0.718	0.469	-0.124	Small
NPRS score	6.58 ± 1.11	0.06 ± 0.33		< 0.001**		
NPQ total score (%)	30.34 ± 8.88	0.83 ± 1.67		< 0.001**		

Data are reported as mean ± standard deviation

*NSNP* Non-specific neck pain, *BMI* Body Mass Index, *NPRS* Numeric pain rating scale, *NPQ* Northwick Park Neck Pain Questionnaire, *CVA* cervicovertebral angle

*Independent t-test *p* < 0.05

**Independent t-test *p* < 0.001

**Fig. 2:**
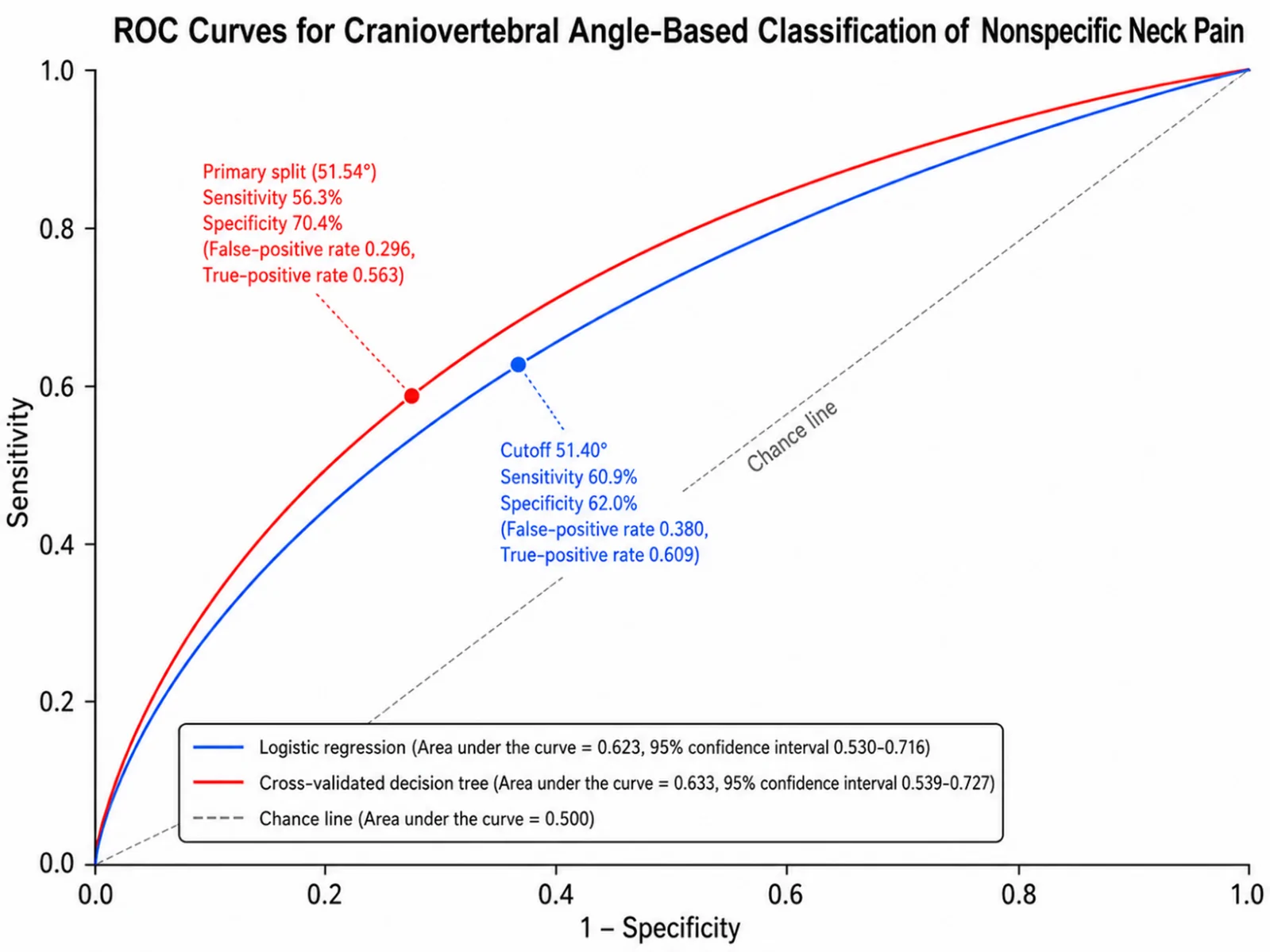
Receiver operating characteristic curves for cervicovertebral angle based classification of nonspecific neck pain

Binary LR analysis was performed to examine the association between CVA and NSNP (Table 2). In the unadjusted model, CVA was significantly associated with NSNP, and the model showed an acceptable fit according to the Hosmer–Lemeshow test (χ² = 6.501, *p* = 0.591). CVA was negatively associated with NSNP (B = − 0.090, SE = 0.038, Wald = 5.682, *p* = 0.017), with an OR of 0.914 (95% CI: 0.849–0.984). This indicates that each 1° increase in CVA was associated with lower odds of NSNP. Conversely, each 1° decrease in CVA was associated with approximately 9% higher odds of NSNP. The unadjusted model showed a Nagelkerke R² of 0.059 and correctly classified 60.7% of cases using the default predicted probability threshold of 0.50, with a sensitivity of 46.9% and specificity of 73.2%. After adjustment for age, height, weight, and BMI, CVA remained significantly associated with NSNP (OR = 0.911, 95% CI: 0.830–0.999, *p* = 0.047). The adjusted model showed a Nagelkerke R² of 0.140, suggesting that the inclusion of demographic covariates improved the explanatory capacity of the model compared with the unadjusted model. However, because height, weight, and BMI are related anthropometric variables, the coefficients of these covariates should be interpreted cautiously. ROC curve analysis was then performed to evaluate the discriminatory ability of CVA for classifying participants with and without NSNP (Fig. 2). The LR-based ROC analysis identified a CVA cutoff of 51.40° based on the Youden index. Using this ROC-derived cutoff, the model showed an accuracy of 61.5%, sensitivity of 60.9%, and specificity of 62.0%. The AUC was 0.623 (SE = 0.049, 95% CI: 0.530–0.716, *p* = 0.014), indicating limited discriminatory ability (Table 3). The sensitivity and specificity values differed from those obtained from the LR classification because the classification used the default predicted probability threshold of 0.50, whereas the ROC analysis used the Youden index-based CVA cutoff.

**Table 2:** Unadjusted and adjusted logistic regression models for the association between craniovertebral angle and nonspecific neck pain

Model	Predictor	OR	95% CI	*p*-value	Nagelkerke *R*²
Unadjusted model	CVA	0.914	0.849–0.984	0.017	0.059
Adjusted model	CVA	0.911	0.830–0.999	0.047	0.140

*CVA* cervicovertevral angle, *OR* odds ratio, *CI* Confidence interval

**Table 3:** Classification accuracy and discriminatory ability of logistic regression and decision tree models

Model	AUC (95% CI)	Accuracy	Sensitivity (95% CI)	Specificity (95% CI)
Logistic regression	0.623 (0.530–0.716)	0.615	0.609 (0.487–0.719)	0.620 (0.503–0.724)
Decision tree	0.645 (0.551–0.739)	0.637	0.563 (0.441–0.677)	0.704 (0.590–0.798)

*AUC* area under the curve, *CI* Confidence interval

**Fig. 3:**
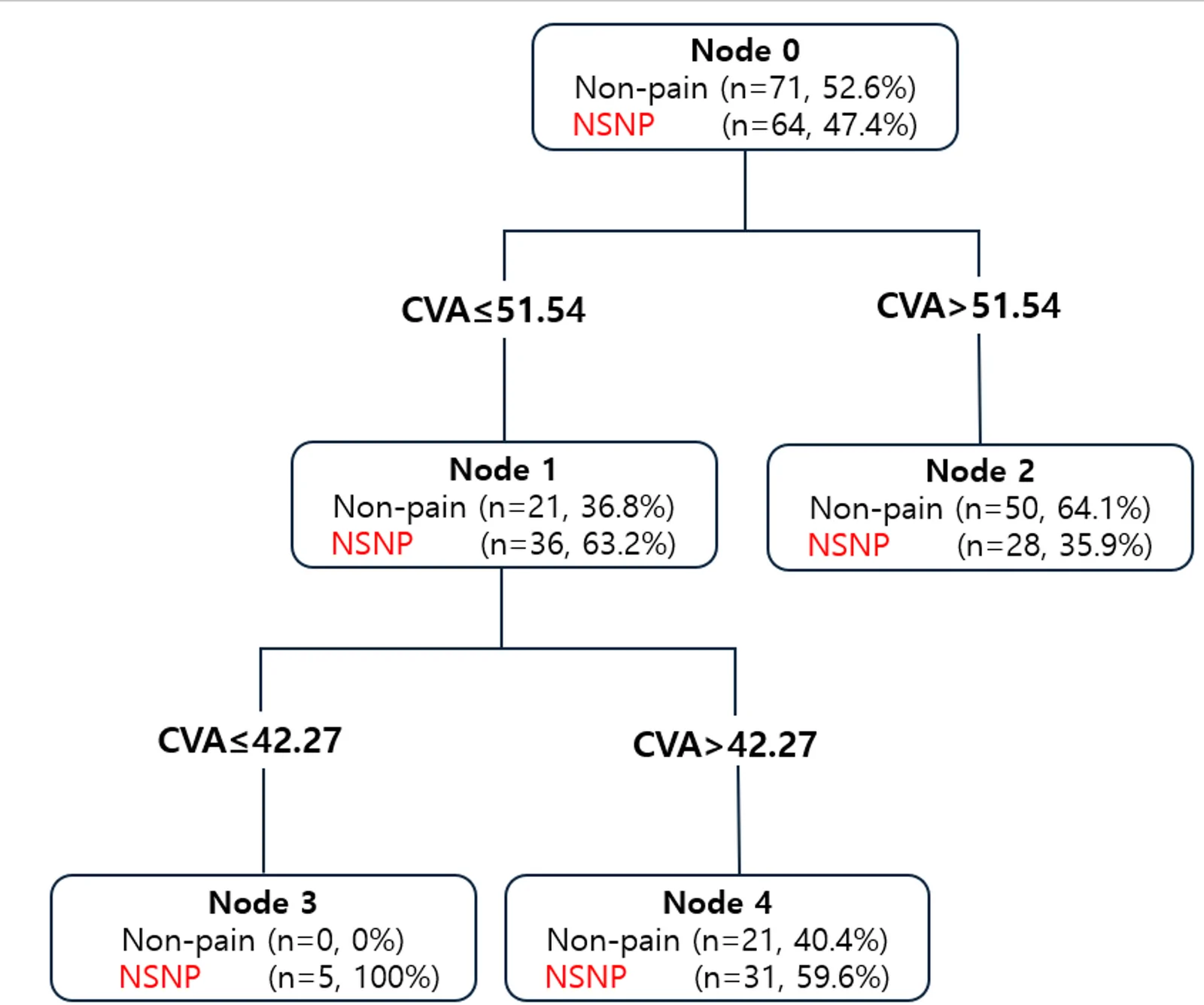
Decision tree analysis for classifying neck pain based on cervicovertebral angle

The DT analysis produced a tree with a maximum depth of two levels and five terminal nodes (Fig. 3). At the first level, the algorithm identified 51.54° as the optimal splitting threshold, partitioning the entire sample into two subgroups: those with CVA ≤ 51.54° (Node 1, *n* = 72) and those with CVA > 51.54° (Node 2, *n* = 63). At the second level, a further split was applied within Node 1, identifying 42.27° as a secondary threshold. The intermediate range of CVA between 42.27° and 51.54° (Node 4) showed an NSNP rate of 59.6%, reflecting substantial within-group variability. The DT model achieved an overall accuracy of 63.7%, sensitivity of 56.3%, and specificity of 70.4%. The AUC was 0.645 (SE = 0.048, 95% CI: 0.551–0.739), indicating slightly higher discriminatory performance than LR, although both models demonstrated poor classification ability.

## Discussion

**Fig. 4:**
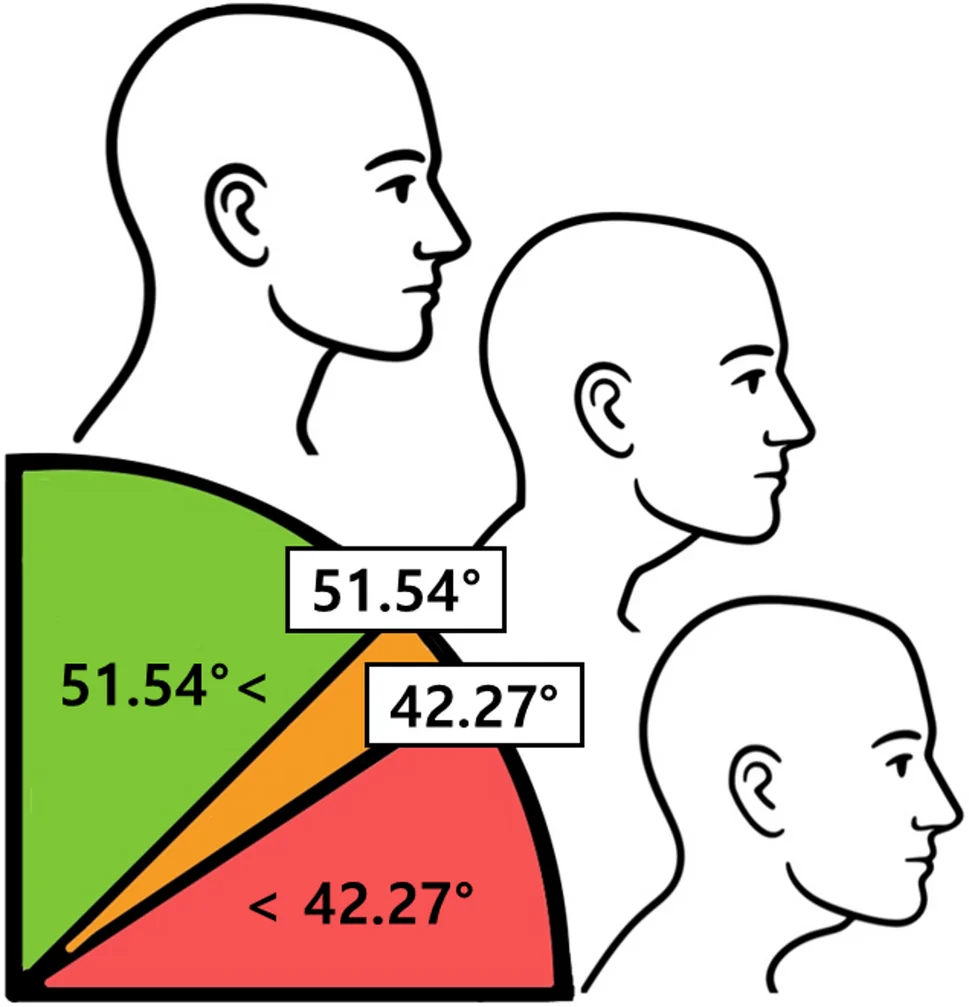
Forward head posture severity categorization using cervicovertebral angle cutoff values

CVA has been widely used by physical therapists and clinicians as a tool to quantitatively screen for FHP, a posture that may be associated with NSNP. Most previous studies have compared CVA between groups with and without NSNP and have reported that, as CVA decreases, the severity of neck pain increases [18, 19, 32, 33]. Nevertheless, a cutoff value for CVA related to neck pain has not yet been identified. This study used LR and DT to evaluate whether CVA is associated with NSNP and identified CVA cutoff values for the first time using statistical modeling. The CVA in the NSNP group (51.16°) was significantly smaller than that in the non-pain group (53.21°), and both models identified a primary cutoff value of approximately 51.4°-51.5° (LR = 51.40°, DT = 51.54°), demonstrating notable consistency across two methodologically distinct approaches. These findings may serve as a statistically derived exploratory reference for supplementary screening (Fig. 4).

However, one of the key findings of this study is that the discriminative performance of CVA is limited when it is used as a single predictor. Both models yielded AUC values below 0.7 (LR: 0.623; DT: 0.645), indicating poor classification ability. This result may reflect the fundamental nature of NSNP as a multifactorial condition. We acknowledge that CVA alone is insufficient to accurately classify individuals with and without NSNP, and the low predictive performance observed in this study was expected given the complexity of the condition. Therefore, the clinical value of the identified cutoff lies not in its diagnostic accuracy, but in its potential utility as a screening reference. A critical finding that may help explain this limitation is the substantial distributional overlap in CVA between the NSNP and non-pain groups. Despite belonging to the NSNP group, many participants presented CVA values much higher than the cutoff value of 51.40°. In contrast, a significant number of participants with CVA values lower than the cutoff value were also found in the non-pain group. This overlap likely contributed to the limited discriminative performance observed in both models. This finding is related to the fact that the present study recruited participants as a single broad group of individuals with NSNP without distinguishing underlying movement impairment patterns. Neck pain can originate from diverse dysfunctional movement patterns, such as lower cervical flexion, lower cervical extension, upper cervical flexion, and upper cervical extension, each involving different biomechanical mechanisms [34–36]. One possible explanation is that different cervical movement or alignment strategies may coexist with lower CVA values. However, such movement pattrens were not directly measured and evaluated in this study, and this interpretation remains hypothetical. Therefore, these findings underscore the need for subgroup-specific analyses in future research.

Regarding the LR model, the OR of 0.914 provides a meaningful quantification of the association between CVA and NSNP, indicating that each one-degree decrease in CVA increases the likelihood of NSNP by approximately 9%. It should be noted that, given the cross-sectional design of this study, the OR represents a measure of association rather than causal risk or prognostic prediction. In addition, the adjusted LR model showed that CVA remained associated with NSNP after controlling for potential confounding variables, suggesting that CVA may have an independent relationship with NSNP. However, the discriminative performance of the adjusted model also remained limited. The overall predictive performance remained low (accuracy = 61.5%; AUC = 0.623). The gap between statistical significance and low classification accuracy shown in this study further emphasizes that the relationship between CVA and NSNP may not be linear.

The classification process of the DT model suggests that the relationship between CVA and NSNP may not be fully explained by a simple linear pattern. In the initial split, Node 0, which included 47.4% of participants with NSNP, was classified at a CVA threshold of 51.54°. Notably, among participants with a CVA greater than 51.54° (Node 2), which may generally be interpreted as a relatively larger or more neutral craniovertebral angle, 35.9% (*n* = 28) were still classified as having NSNP. This finding indicates that NSNP can be present even among individuals with relatively larger CVA values, supporting the multifactorial factors of neck pain and suggesting that CVA alone is insufficient to fully distinguish individuals with and without NSNP. Within the subgroup of participants with a CVA of ≤ 51.54° (Node 1), the DT model identified a secondary split at 42.27°. Participants with CVA values ≤ 42.27° (Node 3) showed a 100% NSNP rate in the present sample. However, this finding should be interpreted cautiously. Because this secondary split was derived from the same dataset without pruning or internal validation/cross-validation, potential overfitting could not be fully excluded. Therefore, the 42.27° threshold should not be regarded as a definitive cutoff value. Rather, it may be considered an exploratory finding suggesting that markedly reduced CVA values could be associated with a higher probability of NSNP. However, the broader intermediate range of 42.27° to 51.54° (Node 4) showed an NSNP rate of only 59.6%, indicating that not all individuals with reduced CVA experienced pain consistently. This variability suggests that, even within the same CVA range, factors such as differing upper and lower cervical movement patterns and muscle function impairments may influence the presence or absence of pain. However, because these factors were not directly assessed in this study, this explanation remains hyphothetical.

Neck pain is influenced by multiple factors, such as sex, age, work environment, and psychological stress [8, 37]. Psychological factors have been found to be the strongest and most consistent influence on the onset and persistence of neck pain [37–39]. Specifically, stress, anxiety, depression, and sleep problems have been shown to be directly related to neck pain intensity and disability levels [37–39]. Therefore, we acknowledge that psychosocial factors are important contributors to NSNP and may improve the explanatory and predictive performance of classification models. In the present study, significant differences were also observed in demographic variables including height, body weight, and BMI, which may represent potential confounding factors. However, psychosocial, occupational, and behavioral variables were not incorporated into the original study design. The primary objective of this study was not to develop a comprehensive multifactorial prediction model, but rather to examine the discriminative utility of CVA as a single static postural metric. Accordingly, the absence of these variables should be considered an important methodological limitation rather than evidence that they are clinically unimportant.

Furthermore, reduced activation and endurance of the deep cervical flexors and extensors have been observed in patients with neck pain, and muscle dysfunction can affect dynamic cervical movement and motor control [40, 41]. In addition to local cervical muscle dysfunction, recent studies support the concept of regional interdependence in chronic neck pain [42, 43]. Previous study suggested that chronic neck pain is associated with reduced strength beyond the cervical region, including pain-free scapulothoracic, shoulder, trunk, and hip regions [42]. Similarly, patients with unilateral chronic nonspecific neck pain have been reported to show strength and range-of-motion deficits on the contralateral side to pain and in other pain-free body regions [43]. These findings suggest that neck pain may be associated with broader impairments in regional motor function rather than being limited to local cervical alignment or isolated cervical muscle performance. In fact, classification accuracy, which was defined as fair when only static CVA data were used, improved to a good level when dynamic movement data such as protraction and retraction of the cervical spine were included [7]. Therefore, the limited predictive ability observed in this study likely reflects both the heterogeneous composition of the NSNP group and the use of CVA alone without incorporating psychosocial, occupational, behavioral, or functional variables. Therefore, clinicians should be cautious when using CVA in clinical settings and future studies should validate the present cutoff value using multivariable models that integrate CVA with psychosocial factors, occupational exposure, pain-related behavior, cervical muscle performance, and dynamic movement variables.

This study had several limitations that should be acknowledged. First, the study subjects were limited to female manufacturing workers in a specific industry; therefore, caution should be exercised when generalizing the cutoff values presented in this study to men or other occupational groups. In addition, although the participants were manufacturing workers, detailed occupational exposure data, such as work posture, repetitive task demands, workstation characteristics, working hours, and specific job type, were not collected. Therefore, this study could not determine how occupational factors may have influenced the relationship between CVA and NSNP. Second, this study relied on CVA as the sole predictor of NSNP. Third, CVA was measured only in the standing position; therefore, it did not reflect CVA in the sitting position, which occupies a large part of daily life. Fourth, the NSNP group was defined relatively strictly as having lower cervical pain and a specific NPRS range (5–8 points); therefore, it may not be representative of all patients with NSNP. Fifth, because no pruning, internal validation, or cross-validation was performed for the DT analysis, the DT-derived cutoff values should be interpreted cautiously as exploratory and potentially sample-specific findings. Finally, this study cannot suggest a causal relationship between CVA reduction and neck pain because of its cross-sectional study design.

## Conclusions

This study identified a primary CVA cutoff value of approximately 51.5° for classifying NSNP among female manufacturing workers. However, the predictive power of CVA alone was limited, indicating that CVA should not be interpreted as a stand alone diagnostic criterion. Therefore, the identified cutoff value should not be interpreted as a definitive diagnostic threshold or as a predictor of future neck pain. Instead, it may provide a preliminary reference value for supplementary clinical screening. Importantly, the generalisability of these findings is limited because the study population consisted exclusively of women employed in the manufacturing industry. Therefore, these findings should be applied cautiously to men, workers in other occupational settings, and broader community populations. Further studies are needed to validate this cutoff value in larger and more diverse samples using multivariable and longitudinal designs.

## Data Availability

The datasets used and/or analysed during the current study are available from the corresponding author on reasonable request.

## Abbreviations

AUC: Area under the curve
BMI: Body mass index
CI: Confidence interval
CRT: Classification and regression tree
CVA: Cervicovertebral angle
DT: Decision tree
FHP: Forward head posture
LR: Logistic regression
NPRS: Numeric Pain Rating Scale
NPQ: Northwick Park Neck Pain Questionnaire
NSNP: Nonspecific neck pain
OR: Odds ratio
ROC: Receiver operating characteristic
